# Metabolomics-Based Pharmacodynamic Analysis of Zhuang Yao Shuang Lu Tong Nao Granules in a Rat Model of Ischemic Cerebral Infarction

**DOI:** 10.1155/2022/8776079

**Published:** 2022-07-05

**Authors:** Yang Zhai, Yangling Chen, Yihui Luo, Xiaoping Mei, Lin Wu, Xueni Mo, Min Zou, Mingzhao Zhou, Yangling Wu, Guangshan Zheng, Peng Yang, Qingyu He, Rui Chen

**Affiliations:** ^1^Graduate School, Guangxi University of Chinese Medicine, China; ^2^Guangxi International Zhuang Medicine Hospital, China; ^3^Department of Neurology, Guangxi University of Chinese Medicine, China; ^4^Faculty of Chinese Medicine Science, Guangxi University of Chinese Medicine, China

## Abstract

This study used a metabolomic approach to reveal changes in the levels of metabolic biomarkers and related metabolic pathways before and after Zhuang Yao Shuang Lu Tong Nao granule (YHT) treatment in rats with cerebral ischemia. The neurological deficit scores were significantly higher in the MCAO_R group than in the NC group, indicating that the mice had significantly impaired motor functions. The YHT group had significantly lower scores than the MCAO_R group, suggesting that YHT significantly improved motor function in rats. TTC staining of the brain tissue revealed that YHT significantly reduced the area of cerebral infarction in the treated rats. The MCAO_R group was better separated from the NC rent, sham, and YHT groups via metabolomic PCA. Moreover, there were significant differences in the differential metabolites between the MACO_R and YHT groups. Eighteen common differential metabolites were detected between the MACO_R and NC groups, MACO_R and sham groups, and MACO_R and YHT groups, indicating that YHT significantly increased the levels of various metabolites in the serum of cerebral ischemic stroke (CIS) rats. Moreover, a total of 23 metabolic pathways were obtained. We identified 11 metabolic pathways with the most significant effects in the bubble plots. In conclusion, from a systems biology perspective, this metabolomics-based study showed that YHT could be used to treat ischemic stroke by modulating changes in endogenous metabolites.

## 1. Introduction

Cerebral ischemic stroke (CIS), also known as acute ischemic cerebrovascular disease, is localized ischemic necrosis of the brain tissue caused by an inadequate blood supply. It is the second leading cause of death worldwide, with a very high mortality rate [1–3]. It is also the leading cause of disability globally, with half of the surviving patients being left with chronic disabilities [4, 5]. The pathophysiology of CIS involves the initial development of irreversibly damaged necrotic tissue cores within the vascular bed, followed by damage around the infarcted area [6–8]. Traditional Chinese medicine (TCM) has a long history of preventing and treating ischemic stroke and promoting the recovery of neurological function after stroke [9]. It is believed that the development of ischemic cerebrovascular disease belongs to the scope of TCM, with the disease showing evidence of a deficiency of the root and the symptoms [10–12].

Zhuang Yao Shuang Lu Tong Nao granule (YHT) is a compound preparation with local characteristics developed by Guangxi International Zhuang Medical Hospital based on the Zhuangtong Drink, which is commonly used in clinical practice. It is mainly composed of four Guangxi characteristic Zhuang medicinal herbs, including *Euonymus 3 otuun*, *Panaxnouogins ng*, *Cinnamomum cassiaPt sl*, and *Polygala fallax H msl*. Its function is to balance qi, invigorate the blood, and unblock the dragon's path; it is commonly used to treat stroke due to qi deficiency and blood stasis [13, 14]. Among them, *Euonymus 3 otuun* has an anti-inflammatory effect [15]; *Panaxnouogins ng* has anti-inflammatory, antioxidant, antiplatelet coagulation, and blood pressure-regulating effects [16]; *Cinnamomum cassiaPt sl* has hypoglycemic, antioxidant, and anticancer activity [17, 18]; and *Polygala fallax H msl* has anti-inflammatory, antioxidant, and neuroprotective effects [19]. In recent years, YHT has been widely used in clinical ischemic stroke for its efficacy in tonifying qi and activating and promoting blood circulation [20–22]. Many studies have been conducted on the use of herbs for the treatment of diseases. *Euonymus laxiflorus* Champ has been found to exhibit several potent biological activities, including anti-NO, enzyme inhibition, and hypoglycemic and antidiabetic effects. Morphine treatment for five days may protect the heart from ischemia-reperfusion (IR) injury [23]. However, no studies have been conducted on the strong medicine Shuanglu Tongue granules.

As a newly developed technology, metabolomics studies the dynamic processes involving metabolites in biological systems after stimulation or disturbance, revealing the metabolic pathways governing biological systems [24–27]. Metabolomics has been successfully used for the diagnosis and classification of early tumors [28], Alzheimer's disease [29], cardiovascular disease [30], cancer [31], and diabetes [32]. Traditional Chinese medicine emphasizes the overall balance of yin and yang in the course of treatment for a disease, which is not limited to a certain body part or organ [33]. Therefore, metabolomics is based on systems thinking, and a holistic view focus on the metabolic profile of endogenous small molecules and the tracking of changes in metabolites to define the state of the body. It is currently one of the most effective ways to evaluate the mechanism of action of Chinese medicine compounds to reflect the functional state of an organism objectively.

At present, there is no research report on the pharmacodynamic effects of ischemic stroke using ultra-high-performance liquid chromatography-quadrupole time-of-flight mass spectrometry from the perspective of its endogenous molecular metabolism. From the change law of endogenous blood metabolites in rats, we used ultra-performance liquid chromatography-quadrupole time-of-flight mass spectrometry (UPLC-MS/MS), principal component analysis (PCA), and partial least squares discriminant analysis (PLS-DA) to reveal the mechanism underlying endogenous molecular metabolism and explain the mode of action of the corresponding active compounds of YHT. Furthermore, we also determined the theoretical significance behind these findings and their broad clinical application prospects.

## 2. Materials and Methods

### 2.1. Materials

Male SD rats (SPF class, 200-230 g) were obtained from Vital River Laboratory Animal Technology Co. Ltd. (Beijing, China; license number SCXK (Hubei) 2017-0012). All rats were housed in a standard environment (humidity, 60 ± 5%, temperature 25 ± 2°C). Animal experiments were conducted at the Animal Experimentation Center, in accordance with the National Laboratory Animal Welfare Guidelines.

### 2.2. Preparation of YHT

YHT included the following components: 15 g *Cinnamomum cassiaPt sl*, 15 g *Autacuylod slanca* (*Thunb.*) *DC*, 15 g *Ctaua gus cun aua* Sieb. Et Zucc., 15 g *Ciutus tuiculaua* Blanco, 10 g Rhizoma Pinelliae, 15 g *Potia cocos* (*Schw.*) *Wolf*, 10 g *Panax notoginseng* (Burk.) F. H. Chen, 20 g *Euonymus 5 otuun* (Turcz.) Hand.–Mazz., 15 g *Zingiber officinale* Roscoe, 5 g Glycyrrhizae, and 20 g *Polygala aureocauda* Dunn. The mixture was immersed in distilled water for 1 h. Reflux extraction was then performed thrice for 1.5 h each time. Afterward, the extract was subjected to water decoction thrice, filtered, and concentrated until it had a syrup-like viscosity and dried in a vacuum drying box at 70°C before obtaining the dry extract.

Quality standards of drug raw materials (medicinal materials) are the following: *Cinnamomum cassiaPt sl*, *Autacuylod slanca* (*Thunb.*) *DC*, *Ctaua gus cun aua* Sieb. Et Zucc., *Ciutus tuiculaua* Blanco, Rhizoma Pinelliae, *Potia cocos* (*Schw.*) *Wolf*, *Panax notoginseng* (Burk.) F. H. Chen, *Euonymus 5 otuun* (Turcz.) Hand.–Mazz., *Zingiber officinale* Roscoe, Glycyrrhiza, and *Polygala aureocauda* Dunn met the requirements of the *Pharmacopoeia of the People's Republic of China* (Volume I, 2020 Edition). The content of corresponding drugs was made sure not to be lower than the specified standards, which were identified by the Department of Traditional Chinese Medicine of the Guangxi University of Traditional Chinese Medicine.

### 2.3. Construction of Animal Models

A rat model of middle cerebral artery occlusion (MCAO) was prepared according to the modified Longa-suture-occluded method. All surgical tools were sterilized under high pressure. After 24 h of fasting, the rats were anesthetized with 10% chloral hydrate (10 mL/kg, i. p.) and fixed on a table. Using a scalpel, the neck skin of the rats was longitudinally cut along the middle of the neck, and the subcutaneous glands and tissues were gradually separated to expose the bilateral common carotid artery (CCA) and external carotid artery (ECA). After ligating the distal end of the ECA and the proximal end of the CCA and clamping the distal end of the ICA with an artery, a small incision was performed in the common carotid artery and the external carotid artery bifurcation. A fishing line (diameter: 0.26 mm) with a rounded tip was inserted into the ICA to the region approximately 2 cm above the bifurcation until resistance was felt. Finally, the ICA was moderately ligated with a spare line, and the neck skin was sutured and sterilized. After 2 h, the plug line was gently pulled out and reperfusion was performed, and the rats were placed in a metabolic cage. The rats in the sham operation group only had their left cerebral artery exposed and did not undergo insertion. During and after the operation, the temperature was maintained at approximately 25-30°C.

### 2.4. Experimental Grouping and Drug Treatment

After the adaptation period, the rats were randomly divided into four groups (*n* = 4 each group). The control group (NC) received normal feeding and had free access to drinking water, gastric perfusion, other volumes of normal saline twice a day (morning/afternoon), and 3 d death after reperfusion. In the model group (MCAO_R), after animal model establishment, the rats received normal feeding and had free access to drinking water, gastric perfusion, other volumes of normal saline twice a day (morning/afternoon), and 3 d death after reperfusion. Rats in the sham operation group (sham) underwent the same procedure as the model group but were not ligated to the artery. After waking up, the rats in this group received normal feeding and had free access to drinking water, gastric perfusion, and other volumes of normal saline twice a day (morning/afternoon) and 3 d death after reperfusion. Finally, in the treatment group (YHT), after establishing the rat MCAO model, the rats were made sober 1 h after anesthesia and were administered intragastrically with Zhuang Yao Shuanglu Tongnao granule water decoction twice a day (morning/afternoon, 1.4 mL/100 g each time) and 3 days after reperfusion. All the rats were administered intragastrically daily for 3 days.

### 2.5. Neurological Function Score

After 2 h of ischemia, the neurological function of the rats was scored using the Longa and Bederson scoring system. This system involves scoring from 0 to 4, with higher scores indicating more severe neurological deficits than lower scores. The specific evaluation criteria for this system are listed in Table 1.

### 2.6. TTC Staining of Brain Tissue

After the cerebellum and olfactory bulbs were removed, the brains of the rats were placed in a refrigerator at −20°C for 5 min. The brains were then cut into four pieces along the optic chiasm and 2 mm along the optic nerve. The brain slices were stained in 2% TTC solution and placed in a water bath at 37°C. After 20 min, the brain slices were removed, washed in normal saline three times, and fixed in a 10% formaldehyde solution for 24 h for imaging.

### 2.7. Preparation of Serum Samples

After 24 h of reperfusion, 2 mL of the orbital blood was collected and placed in an EP tube. After incubation for 30 min, the supernatant was centrifuged at 4000 rpm, 4°C for 10 min. The supernatant was then extracted using a pipette gun and transferred to an EP tube. After labeling, the supernatant was placed in a refrigerator at −80°C. Finally, the metabolomic parameters of the collected serum were analyzed.

### 2.8. UPLC-MS/MS Analysis

All cryopreserved sera require additional treatment before UPLC-MS/MS analysis. First, serum samples were removed from a −80°C refrigerator and were thawed slowly on ice. Next, 100 *μ*L aliquots were placed in 1.5 mL centrifuge tubes, 300 *μ*L of methanol was added to the serum aliquots, and the mixture was vortexed for 30 s. The solutions were then incubated for 1 h at −40°C. The solutions were vortexed again for 30 s and then incubated for 30 min at 4°C. Then, the solutions were centrifuged at 4°C, 12000 rpm for 15 min, the supernatants were collected in centrifuge tubes, and the tubes were incubated for 1 h at −40°C. Finally, the collected supernatants were centrifuged again at 4°C, 12000 rpm for 15 min, and 200 *μ*L of the clear supernatant was collected. This, including 5 *μ*L of an internal standard (140 *μ*g/mL dichlorophenylalanine), was subjected to UPLC/MS-MS analysis.

### 2.9. Chromatographic Separation Conditions

Column temperature is 40°C, flow rate is 0.65 mL/min, mobile phase A is water with 10 mM ammonium formate, mobile phase B is acetonitrile with 10 mM ammonium formate, injection volume is 3 *μ*L, and automatic injector temperature is 4°C (Table 2). The mass spectrometry conditions are as follows: ESI+; heater temperature is 300°C; sheath gas flow rate is 45 arb; aux gas flow rate is 15 arb; sweep gas flow rate is 1 arb; spray voltage is 3.0 kV; capillary temperature is 350°C; S-Lens RF level is 30%.

### 2.10. Data Processing and Analysis

Feature extraction was performed, and the data obtained were preprocessed using Compound Discoverer software (Thermo) and then normalized and edited into a two-dimensional data matrix using Excel 2010 software, including the retention time (RT), compound molecular weight (compMW), observations (samples), and peak intensity.

To reveal the differences in metabolites among the different groups, the data were input into SIMCA software (v14.1, Sartorius Stedim data analytics AB, Umea, Sweden) and subject to a series of multivariate pattern recognition analyses, including PCA, PLS-DA, and orthogonal partial least squares discriminant analysis (OPLS-DA). At the same time, different metabolites were screened based on the principle regarding the *P* value of Student's *t*-test (*P* ≤ 0.05) and the Variable Importance in the Projection (VIP ≥ 1), the first main component of the OPLS-DA model. The results were visualized using volcano plots. In addition, hierarchical clustering (HCA) and correlation analysis of the metabolites were used to reveal the relationship between samples and metabolites. Finally, the biological significance of the metabolites was explained through a functional analysis of the metabolic pathways involved.

## 3. Results

### 3.1. Effects of YHT on Neurological Function and Cerebral Infarction in Mouse Model

**Figure 1:**
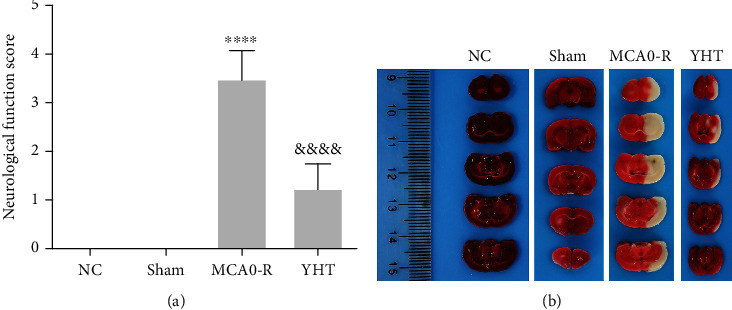
Effect of YHT on neurological function and cerebral infarction in the MCAO model rats: (a) neurological function scores; (b) TTC staining to observe the condition of brain tissue in mice. ^∗∗∗∗^*P* < 0.0001 compared with the NC group; ^&&&&^*P* < 0.0001 compared with the MCAO_R group.

Neurological function scores were obtained for each group of mice (Figure 1(a)). It was found that the scores in the MCAO_R group were significantly higher (*P* < 0.05) than those in the NC group, indicating that the mice had increased walking difficulty toward the contralateral side, and the model was successfully constructed. Moreover, the neurological function scores were significantly lower in the YHT group than in the MCAO_R group (*P* < 0.05). We then observed the brain tissue conditions of the mice using TTC staining (Figure 1(b)). There was no obvious infarction in the brain tissues of rats from the NC and sham groups, while those in the MCAO_R group had severe brain tissue infarction. The infarcts in the YHT group were significantly reduced compared with those in the MCAO_R group. These results indicate that YHT can relieve the symptoms of middle cerebral artery occlusion by reducing cerebral infarction.

### 3.2. Principal Component Analysis and Partial Least Squares Discriminant Analysis of the Metabolites Present in the Rat Serum

**Figure 2:**
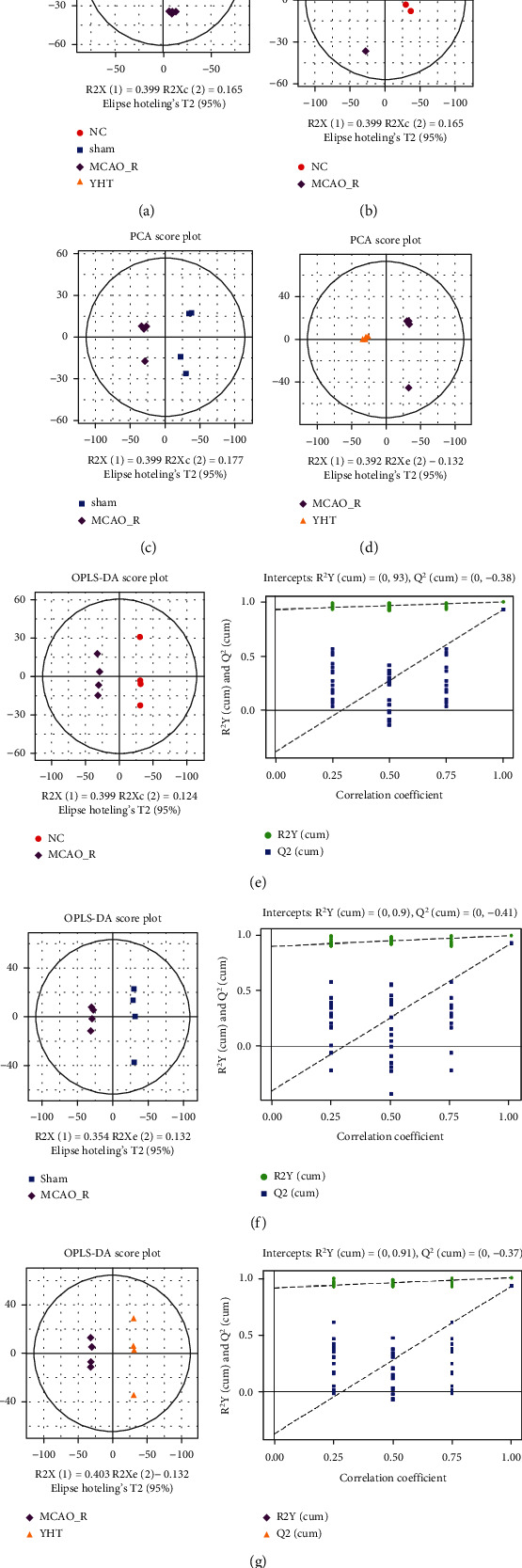
Principal component analysis and partial least squares discriminant analysis of the components of mouse serum: (a) principal component analysis for each group of rats; (b) principal component analysis between the NC and MCAO_R groups; (c) principal component analysis between the sham and MCAO_R groups; (d) principal component analysis between the MCAO_R and YHT groups; (e) partial least squares discriminant analysis between the NC and MCAO_R groups; (f) partial least squares discriminant analysis between the sham and MCAO_R groups; (g) partial least squares discriminant analysis between the MCAO_R and YHT groups.

PCA was performed on the sera of rats to observe the overall distribution trends of metabolites in each group and the changes in these trends after YHT treatment. The results showed that the MCAO_R group was distributed below the circle and was separated from the other groups of mice, while the NC and sham groups had a crossover distribution, indicating that the metabolites in the two groups did not differ significantly. Since no ischemia experiments were performed in these groups, the effect of traumatic manipulation on cerebral ischemia metabolism was not significant. The YHT group was well separated from the MCAO_R group, indicating that YHT could relatively improve cerebral ischemia (Figure 2(a)). To observe the effect of YHT treatment on serum metabolites more clearly, the MCAO_R group was subjected to PCA separately from the NC group (Figure 2(b)), the sham group (Figure 2(c)), and the YHT group (Figure 2(d)), all of which were found to be distinguishable.

To further investigate the therapeutic mechanism of YHT, a discriminant model was developed in this study between the MCAO_R and NC groups, the MCAO_R and sham groups, and the MCAO_R and YHT groups. The score charts of the MCAO_R and NC groups are shown in Figure 2(e). The MCAO_R and NC groups were significantly located on both sides, indicating significant differences in the metabolic components between the two groups. The model interpretation capability parameter (*R*^2^*Y*) was 0.99, and the other prediction capability parameter (*Q*^2^) was 0.93, indicating that the model has a good ability to distinguish and predict. According to the permutation test, all *R*^2^*Y* values were higher than the *Q*^2^ values, and their values were close to 1. This indicates an absence of overfitting in the model, so it could be used to screen the differential markers. For example, the *R*^2^*Y* and *Q*^2^ values were 0.99 and 0.93, respectively, when comparing the MCAO_R group with the sham group (Figure 2(f)); the *R*^2^*Y* values were higher than the *Q*^2^ values according to the permutation test. Similarly, between the MCAO_R group and the YHT group, the *R*^2^*Y* and *Q*^2^ values were 1.0 and 0.93, respectively (Figure 2(g)). The permutation test results also showed that the model was similar to the above model, so it can also be used to screen for differential markers.

### 3.3. Identification of Differential Serum Metabolites

**Figure 3:**
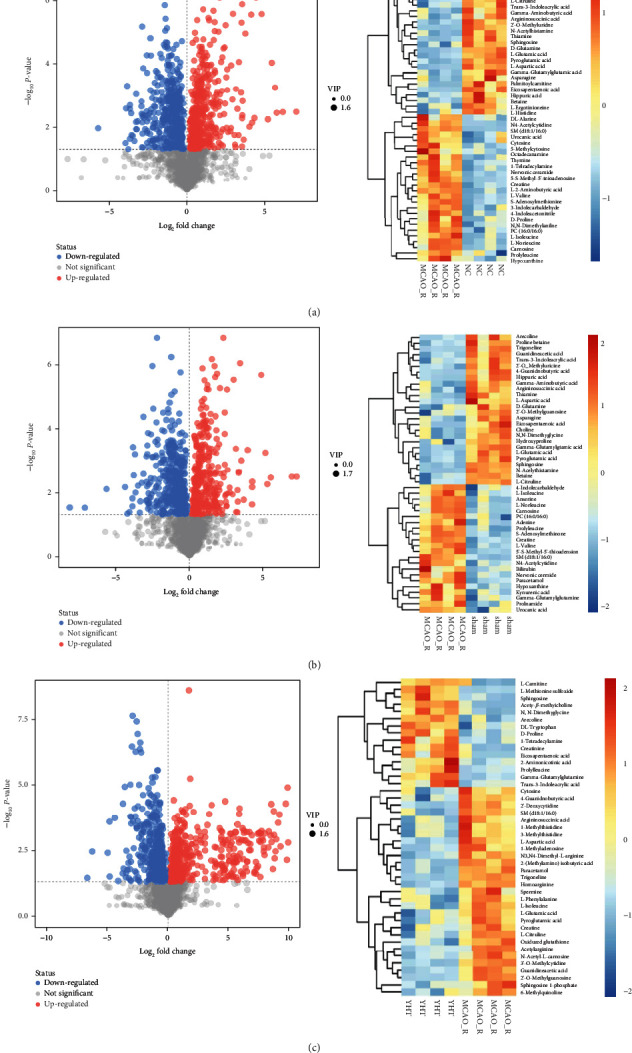
Identification of serum differential metabolites: (a) identification of heterogeneous metabolites between the NC and MCAO_R groups; (b) identification of heterogeneous metabolites between the sham and MCAO_R groups; (c) identification of heterogeneous metabolites between the MCAO_R and YHT groups; (d) identification of heterogeneous metabolites between the MCAO_R and YHT groups; (e) identification of heterogeneous metabolites between the MCAO_R and YHT groups.

After analysis, it was confirmed that the model could be used to screen for differential markers. Differential metabolites were screened using the cardinality criterion of Student's *t*-test (*P* < 0.05), while Variable Importance in the Projection (VIP) > 1 was the first principal component of the OPLS-DA model. We visualized the results of screening the differential metabolites using a volcano plot and then performed a hierarchical cluster analysis on the characteristics of the obtained differential metabolites. Each point in the volcano plot represents a metabolite, while the scatter color represents the final screening result. Significantly upregulated metabolites are shown in red, significantly downregulated ones are shown in blue, and nonsignificantly different ones are shown in gray. The MACO_R group was analyzed separately from the other groups (NC, sham, and YHT) for differential metabolite levels. In the analysis of the MACO_R group versus the NC group (Figure 3(a)), the changes in the differential metabolites were opposite. Similarly, in the comparative analysis between the sham group (Figure 3(b)) and the YHT group (Figure 3(c)), an opposite trend was found in the changes in differential metabolites compared to the MACO_R group. Eighteen common differential metabolites were detected between the MACO_R and NC groups, the MACO_R and sham groups, and the MACO_R and YHT groups (Table 3). These results suggest that YHT can improve cerebral ischemia in rats by regulating the metabolism of endogenous substances in the serum, with good therapeutic effects.

### 3.4. Metabolic Pathway Analysis

**Figure 4:**
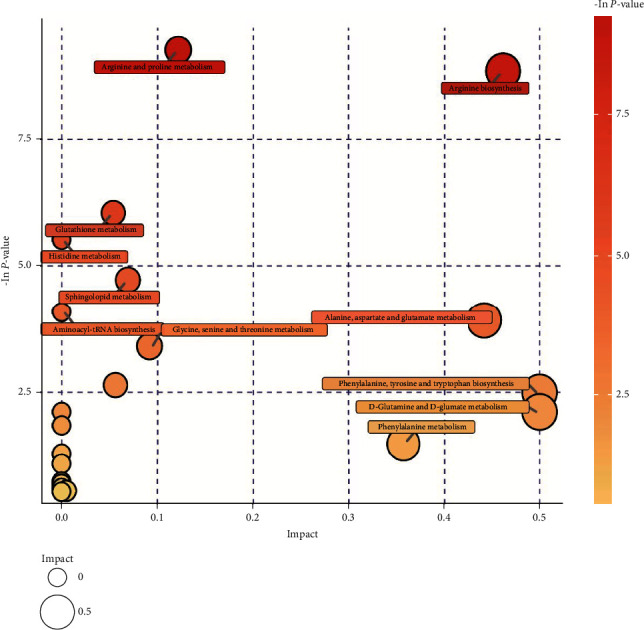
Analysis of metabolic pathways.

Compositional analysis and screening identified the differential endogenous metabolites in rat serum. The MetaboAnalyst 4.0 and KEGG databases were used to analyze the relevant metabolic pathways of these metabolites. A total of 23 metabolic pathways were identified (Table 4), including the metabolism of arginine and proline; biosynthesis of arginine; metabolism of glutathione; metabolism of histidine; metabolism of sphingomyelin; biosynthesis of aminoacyl tRNA; metabolism of alanine, aspartate, and glutamate; metabolism of glycine, serine, and threonine; metabolism of *β*-alanine; biosynthesis of phenylalanine, tyrosine, and tryptophan; metabolism of nitrogen; metabolism of d-glutamine and d-glutamate; biosynthesis of valine, leucine, and isoleucine; metabolism of phenylalanine; metabolism of butyric acid; metabolism of nicotinic acid and nicotinamide; biosynthesis of pantothenic acid and CoA; metabolism of porphyrins and chlorophyll; metabolism of acetaldehyde and dicarboxylic acids; biosynthesis of unsaturated fatty acids; metabolism of pyrimidines; and degradation of valine, leucine, and isoleucine. Using bubble plots, we then identified 11 metabolic pathways with the most significant effects (Figure 4): arginine and proline metabolism; arginine biosynthesis; glutathione metabolism; histidine metabolism; sphingolipid metabolism; aminoacyl-tRNA biosynthesis; alanine, aspartate, and glutamate metabolism; glycine, serine, and threonine metabolism; alanine, aspartate, and glutamate metabolism; phenylalanine, tyrosine, and tryptophan biosynthesis; d-glutamine and d-glutamate metabolism; and phenylalanine metabolism.

## 4. Discussion

CIS is the second leading cause of death [34]. It causes neurological impairments leading to various complications, including Parkinson's disease, chorea, vision loss, and memory loss, and has a mortality rate of up to 30% [35]. Its pathogenesis is unclear, and the available drugs for this disease are not ideal. Therefore, finding effective ways to treat and prevent stroke is essential. Many clinical and experimental studies have demonstrated that YHT has multiple biological activities, such as vasodilation, antioxidant activity, reducing blood viscosity, improving microcirculation, and promoting the proliferation of endogenous neural stem cells and neural regeneration after ischemic stroke [20].

Many studies have shown that the MCAO model is consistent with human stroke pathogenesis and has the advantages of good reproducibility and stability, thus providing a powerful tool for ischemic encephalopathy research and drug screening [34]. This study revealed changes in the levels of metabolic biomarkers and related metabolic pathways before and after YHT treatment in cerebral ischemic rats. The results revealed that YHT treatment significantly reduced neurological function scores, indicating that it restored CIS-induced motor impairment, improved brain damage, and reduced the area of cerebral infarction in a rat model of ischemic cerebral infarction. Moreover, metabolomic PCA revealed that the YHT group was well-separated from the MCAO_R group. Analysis of differential metabolites in mouse serum also revealed that YHT significantly improved various metabolites in the serum of CIS rats.

Eleven metabolic pathways with the most significant effects were identified using bubble plots. In the amino acid metabolism pathway, glutamine produces glutamic acid and ammonia through glutaminase. It then enters the TCA cycle through glutamic acid dehydrogenase, aspartate aminotransferase, and alanine aminotransferase to produce *α*-ketoglutaric acid. Alternatively, it can be completely oxidized to CO_2_ or partially oxidized to produce alanine, aspartate, or lactic acid. L292 cells mainly convert glutamine to aspartic and glutamic acids. As a neurotransmitter, when the amount of glutamate in the intercellular space increases, the concentration of excitatory amino acids increases. This, in turn, activates NMDA receptors, opens calcium channels, stimulates a large number of free radicals, activates postsynaptic glutamate receptors, activates enzymes, and triggers signaling cascade reactions. Glutamate and aspartate may also produce neurotoxins. This reaction cascade allows oxygen radicals and other messengers to activate inflammatory cytokines and enzymes to trigger an inflammatory response in activated microglia. Inflammation produces free radicals, which can lead to a vicious cycle. Arginine is an amino acid wherein proline is an alternative precursor for its synthesis. Arginine can be synthesized from citrulline through the metabolism of glutamine and proline. Arginine exerts antioxidant effects by reducing the production of superoxide anions in the body. It can also accelerate the ornithine cycle, in which carbamyl phosphate participates in urea synthesis, increases the formation of urea, and accelerates the removal of ammonia. A deficiency in arginine leads to the accumulation of carbamate phosphate, increasing the production of whey acid. Aminoformyl phosphate reacts with ornithine to form citrulline and phosphoric acid, becomes converted to arginine, and cleaves to ornithine and urea. Ornithine can be interconverted with arginine, proline, and glutamic acid and transferred to *α*-ketoic acid, glyoxylic acid, and ornithine dehydroxylase to produce butylenediamine. These products can be used as precursors to further synthesize polyamines. Ornithine and citrulline can reduce the negative nitrogen balance and improve immunity, respectively.

Lipids also have essential biological functions. Metabolic disorders can cause inflammation, nerve cell damage, degeneration, and even death, causing irreversible damage to the brain. The glycerol phospholipid pathway plays an important role in promoting blood circulation and preventing blood stasis. Phospholipids and sphingolipids are essential components of the brain lipids. Glycerolipids are the most widely distributed phospholipid components in the cell membrane, and their content is relatively high. Sphingomyelin is the main lipid in myelin. Cell membranes in the brain can quickly respond to changes in the environment, which requires the continuous regulation of membrane phospholipids and sphingolipids. When lipid metabolism is disturbed, excitatory amino acid receptors are stimulated, and calcium channels and associated phospholipases are activated. Phospholipase hydrolyzes phosphatidylcholine and phosphatidylethanolamine on the cell membrane into diglyceride, phosphatidic acid, arachidonic acid, and other chemical moieties. Simultaneously, arachidonic acid activates sphingolipase to hydrolyze sphingolipids into ceramide. Ceramide induces neuronal apoptosis by inhibiting the mitochondria and releasing cytochrome. Under the action of phospholipase, arachidonic acid produces phosphatidylglycerol, phosphatidylserine, phosphatidylinositol, and phosphatidylethanolamine, all of which participate in cell membrane reconstruction. In summary, the main action of YHT is carried out through the seven metabolic pathways mentioned above, while the mechanism of activating blood circulation and resolving blood stasis may be more similar to the changes in amino acid and phospholipid metabolic pathways. This needs to be explored further in future research.

## 5. Conclusion

In conclusion, YHT promoted the recovery of neurological function and significantly reduced the area of brain infarction in MCAO model rats. Metabolic studies have suggested that the apparent neuroprotective mechanism of prolotherapy in a focal cerebral ischemia model is related to the changes in the levels of endogenous metabolites. However, the bioactive compounds responsible for the anti-ischemic effect of YHT need to be verified in further studies.

## Figures and Tables

**Table 1:** Neurological function scoring criteria.

Performance	Rating/score
*Longa rating*	
No defects	0
Difficulty in full extension of the contralateral forelimb	1
Rotate to opposite side	2
Drop to the opposite side	3
Inability to walk spontaneously	4
*Bederson rating*	
No defects	0
It is difficult to fully extend the contralateral forelimb when lifting the tail	1
Rotate to opposite side	2
Reduced contralateral push resistance (and forelimb flexion) without circling	3
Reduced contralateral push resistance (and forelimb flexion)	4

**Table 2:** The gradient of mobile phase.

Time (min)	Flow rate (mL/min)	*A* (%)	*B* (%)
0.00	0.65	5	95
0.5	0.65	5	95
9.5	0.65	35	65
11.50	0.65	60	40
13.50	0.65	60	40
13.60	0.65	5	95
16.00	0.65	5	95

**Table 3:** Differential metabolites in serum samples.

Number	Metabolites	Molecular formula	RT (min)	*P* value			Fold change		
				MACO_R/NC	MACO_R/sham	MACO_R/YHT	MACO_R/NC	MACO_R/sham	MACO_R/YHT
1	Creatine	C_4_H_9_N_3_O_2_	10.795	<0.001	<0.001	0.043	1.379	1.465	0.939
2	L-Citrulline	C_6_H_13_N_3_O_3_	11.832	0.001	<0.001	0.005	0.705	0.742	0.717
3	L-Isoleucine	C_6_H_13_NO_2_	8.744	0.008	0.012	0.006	1.257	1.265	0.668
4	SM(d18:1/16:0)	C_39_H_79_N_2_O_6_P	5.104	0.004	<0.001	0.002	1.238	1.268	0.723
5	Trigonelline	C_7_H_7_NO_2_	9.499	0.024	0.019	<0.001	0.265	0.221	0.179
6	L-Glutamic acid	C_5_H_9_NO_4_	12.589	<0.001	<0.001	0.013	0.410	0.460	0.638
7	4-Guanidinobutyric acid	C_5_H_11_N_3_O_2_	10.878	0.037	0.018	0.006	0.369	0.307	0.726
8	Guanidineacetic acid	C_3_H_7_N_3_O_2_	10.949	0.012	0.001	0.001	0.445	0.384	0.224
9	Pyroglutamic acid	C_5_H_7_NO_3_	12.540	<0.001	0.001	0.026	0.425	0.477	0.670
10	Prolylleucine	C_11_H_20_N_2_O_3_	11.366	0.026	0.001	0.045	1.499	2.079	1.770
11	L-Aspartic acid	C_4_H_7_NO_4_	12.746	<0.001	0.023	0.010	0.305	0.250	0.352
12	Eicosapentaenoic acid	C_20_H_30_O_2_	2.468	0.005	0.015	0.039	0.148	0.165	4.080
13	Sphingosine	C_18_H_37_NO_2_	5.079	0.005	<0.001	0.002	0.238	0.222	2.216
14	Argininosuccinic acid	C_10_H_18_N_4_O_6_	13.513	0.017	0.004	0.023	0.535	0.555	0.432
15	Arecoline	C_8_H_13_NO_2_	10.695	0.040	0.028	0.002	0.180	0.156	2.200
16	trans-3-Indoleacrylic acid	C_11_H_9_NO_2_	2.538	0.004	0.003	0.043	0.353	0.311	3.577
17	Paracetamol	C_8_H_9_NO_2_	11.158	0.001	0.001	<0.001	2.013	1.780	0.151
18	2′-O-Methylguanosine	C_11_H_15_N_5_O_5_	6.812	0.030	0.011	0.006	0.700	0.691	0.274

**Table 4:** Metabolic pathway analysis.

Pathway	Raw *P*	-ln(*P*)	FDR	Impact
Arginine and proline metabolism	<0.001	9.258	0.006	0.122
Arginine biosynthesis	<0.001	8.846	0.006	0.462
Glutathione metabolism	0.002	6.037	0.067	0.054
Histidine metabolism	0.004	5.515	0.085	0.000
Sphingolipid metabolism	0.009	4.723	0.149	0.069
Aminoacyl-tRNA biosynthesis	0.017	4.084	0.236	<0.001
Alanine, aspartate, and glutamate metabolism	0.020	3.922	0.238	0.442
Glycine, serine, and threonine metabolism	0.033	3.405	0.349	0.092
Beta-alanine metabolism	0.071	2.642	0.665	0.056
Phenylalanine, tyrosine, and tryptophan biosynthesis	0.082	2.498	0.691	0.500
Nitrogen metabolism	0.121	2.113	0.846	<0.001
D-Glutamine and D-glutamate metabolism	0.158	2.113	0.846	0.500
Valine, leucine, and isoleucine biosynthesis	0.158	1.846	1.000	<0.001
Phenylalanine metabolism	0.228	1.481	1.000	0.357
Butanoate metabolism	0.276	1.287	1.000	<0.001
Nicotinate and nicotinamide metabolism	0.276	1.287	1.000	<0.001
Pantothenate and CoA biosynthesis	0.336	1.090	1.000	<0.001
Porphyrin and chlorophyll metabolism	0.478	0.739	1.000	<0.001
Glyoxylate and dicarboxylate metabolism	0.500	0.693	1.000	<0.001
Biosynthesis of unsaturated fatty acids	0.542	0.613	1.000	<0.001
Pyrimidine metabolism	0.571	0.560	1.000	0.005
Valine, leucine, and isoleucine degradation	0.581	0.544	1.000	<0.001

## Data Availability

The data used to support the findings of this study are included within the article.
